# Bacteriophages: an underestimated role in human and animal health?

**DOI:** 10.3389/fcimb.2014.00039

**Published:** 2014-03-28

**Authors:** Marianne De Paepe, Marion Leclerc, Colin R. Tinsley, Marie-Agnès Petit

**Affiliations:** ^1^Institut National de la Recherche Agronomique, Micalis, UMR 1319Jouy en Josas, France; ^2^Agroparistech, Micalis, UMR 1319Jouy en Josas, France

**Keywords:** virome, digestive tract, community shuffling, biological weapon, horizontal transfer

## Abstract

Metagenomic approaches applied to viruses have highlighted their prevalence in almost all microbial ecosystems investigated. In all ecosystems, notably those associated with humans or animals, the viral fraction is dominated by bacteriophages. Whether they contribute to dysbiosis, i.e., the departure from microbiota composition in symbiosis at equilibrium and entry into a state favoring human or animal disease is unknown at present. This review summarizes what has been learnt on phages associated with human and animal microbiota, and focuses on examples illustrating the several ways by which phages may contribute to a shift to pathogenesis, either by modifying population equilibrium, by horizontal transfer, or by modulating immunity.

## Introduction

Parallel to the development of metagenomics studies on microbial ecosystems (Lepage et al., 2013), specific approaches for the description of the viral fraction of these ecosystems have emerged in the recent years, resulting in the description of various ecosystem “viromes.” These observations have highlighted the remarkable importance of viruses in all studied microbial ecosystems since their description in the Sargasso sea samples (Angly et al., 2006). Their high diversity was in particular described in microbial communities living in symbiosis with the human body such as the digestive tract (Reyes et al., 2010; Minot et al., 2011), the human saliva (Pride et al., 2012), the respiratory tract (Willner et al., 2009) or the skin (Foulongne et al., 2012), but also animal microbiota, such as in the cow rumen (Berg Miller et al., 2012), or in the pig digestive tract (Shan et al., 2011). Interestingly, such metagenomic approaches allow the recovery of viruses from the three kingdoms of life, which are seldom looked at together. In most of the above mentioned cases, the viral fraction is essentially composed of viruses of bacteria, also designated as bacteriophages (or phages for short). This appears not to be the case for the human skin virome, where eukaryotic viruses were reported to be dominant, but the sample preparation did not include the steps needed to recover fully the bacterial viruses (Foulongne et al., 2012). Indeed, critical to the appropriate interpretation of virome studies is the technique used to purify virus particles away from other DNA and RNA sources, and extract their genetic content (Roux et al., 2013). A recent survey exposes the key technological issues and summarizes what has been learnt by applying such techniques to the human gut viral fraction (Reyes et al., 2012). These various virome studies have naturally fostered a renewed interest for the possible impact of bacterial viruses on ecosystems.

The influence of phages on bacterial ecosystems is manifold, and for a comprehensive reading on their influence in shaping the mammalian gut microbiota, readers are referred to a recent review (Mills et al., 2013). Here we more specifically address the possible contribution of phages to the shift from health to disease in humans and animals with emphasis on the digestive tract ecosystem. The gut microbiota is a very complex microbial ecosystem, comprised of more than 500 species living in eubiosis with their host; i.e., with mutual benefit. Over the past decade, an increasing number of studies have indicated that changes in the abundance of bacterial species, or imbalance of the dynamic equilibrium in bacterial composition, are linked to several human disorders. A contribution of phages to the shift from health to disease could be related to their potential involvement in dysbiosis, a term which designates the rupture of eubiosis (see several definitions in Box 1). However, up to now, very few examples of phage contribution to a change in microbiota composition have been reported. Here, we present concepts and related data useful to understand this emerging field of research. Much more is known on the contribution of phages to disease via their introduction into the bacterial genome, and expression of phage genes dramatically changing bacterial phenotypes. Such modification is known as lysogenic conversion, and famous examples include toxin genes such as the Panton-Valentine, cholera, Shiga- and diphtheria toxins. Combined with the capacity of temperate phages to spread from one bacterial host to another, it can lead to the emergence of new pathogenic bacterial strains (Brussow et al., 2004; Tinsley et al., 2006; Fortier and Sekulovic, 2013). A recent and dramatic case of such emergence is the *Escherichia coli* O104:H4 strain which acquired a Shiga-toxin encoding phage, and caused an outbreak in Germany in 2011 (Muniesa et al., 2012). Finally, phages can also impact the host immune response, either directly or by modifying the antigenicity of bacteria.

Box 1 **Glossary**

**Microbiota**: A microbial community, including bacteria, archaea, eukarya, and viruses, which occupies a given habitat.

**Metagenomics**: The study of metagenomes, genetic material recovered directly from environmental samples. Metagenomics is the genomic analysis (analysis of the entire DNA in an organism) applied to all the microorganisms of a microbial ecosystem without previous identification. Metagenomics is an emerging field encompassing culture-independent studies of the structures and functions of microbial communities and their interactions with the habitats they occupy to understand their biological diversity.

**Dysbiosis**: Alteration of the microbiota in comparison to the normal, healthy state. Dysbiosis refers to a condition of microbial imbalances compared with the “eubiosis” condition, a state of equilibrium between bacterial symbionts and pathobionts. Dysbiosis is mostly reported in the digestive tract or on the skin, but can also occur on any exposed surface or mucous membrane. Dysbiosis can be observed at several analytical levels, from diversity to compositional or functional imbalances.

**Symbiosis**: from the Greek “sym”—together—and “bioo”-living-, designates a stable and intimate association between two or more organisms belonging to different species. The organisms are designated as symbionts, the larger one can also be named the host. A symbiosis does not include necessarily the idea of reciprocal benefit, which is designated by mutualism.

**Pathobionts**: strains of the gut microbiota that are harmless in healthy individuals but become pathogen upon major shifts such as antibiotherapy, or the weakening of host immunity. All pathobionts are opportunistic pathogens, but conversely, some opportunistic pathogens may not be part of the healthy gut microbiota. The term was coined in 2009 by Round and Mazmanian (2009).

**Monoxenic mice**: Germ-free mice maintained in sterile incubators that have been fed with a single bacterial species, that establishes a symbiosis with the gut.

### Abundance, diversity, localization of phages in the digestive tract

#### Lysis and lysogeny

**Figure 1:**
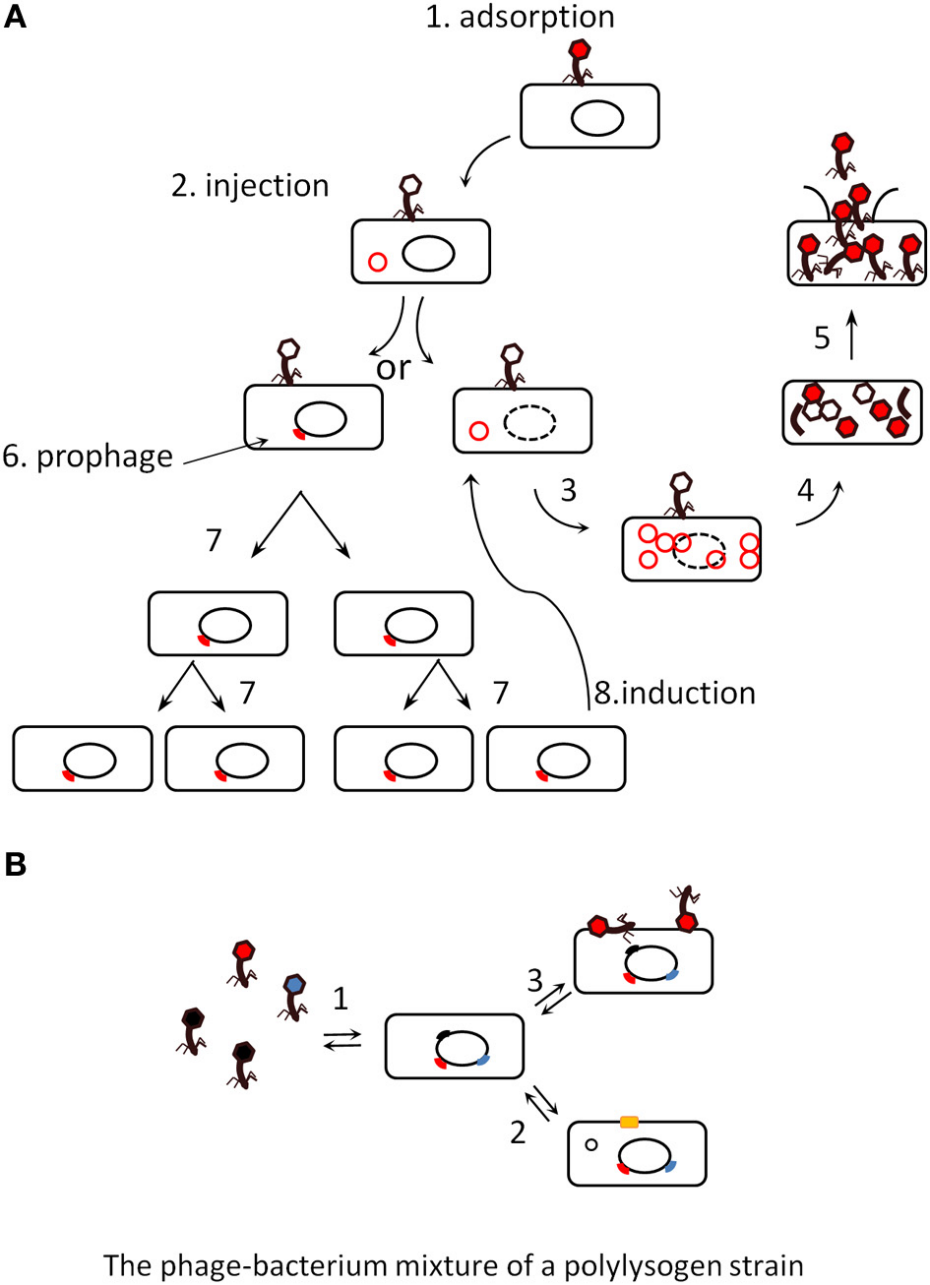
**(A)** The lytic and lysogenic life cycles of phages (scheme taken from Dr Gary Kaiser, with permission). Virulent phages perform only lytic cycles (steps 1 to 5), they adsorb to the bacterial surface (1), inject their DNA (2), replicate it (3), produce capsid proteins that assemble together (4), and finally lyse their host (5). Temperate phages can proceed similarly or shift to a lysogenic cycle (6), where they enter a silent stage of prophage (usually integrated into the bacterial genome), and are replicated passively by the bacterial machinery (7). Either a rare stochastic event or a stress induces the prophage (8), which then comes back into a lytic cycle. **(B)**. The various states of a poly-lysogen strain. Most bacterial strains are poly-lysogens, they host more than one prophage. As a consequence, a population may differentiate into various states: it can produce (1) background levels of virions, (2) excise some prophage and reveal a new phenotype by restoration of the gene interrupted by the prophage [producing for instance a surface protein, yellow square (Rabinovich et al., 2012)], (3) adsorb virions and use them as virulence factors (Mitchell et al., 2007).

Phages essentially belong to two categories, *virulent* and *temperate*. The virulent phage life cycle consists in replicating in, and then lysing their bacterial host (Figure 1A, steps 1–5). Temperate phages can alternate between two life styles: they either lyse their host, like virulent phages, or establish a symbiosis with it, the so-called *lysogeny* state (Figure 1A steps 6 and 7), by entering into the bacterial genome and expressing only a small subset of their genes (among which, for instance, the above mentioned toxins). Upon infection in a given bacterial population, the fraction of temperate phages entering into lysogeny varies with the bacterium physiology, and is generally low, compared to the fraction going into lysis. In the condition of lysogeny, the phage is named “*prophage*,” and the bacterium hosting the phage is said to be *lysogenic* for this phage. The study of naturally occurring signals triggering the shift back from the symbiosis life style to the lytic one (Figure 1A, step 8) is an ongoing field of research. Known signals of prophage induction are mainly related to DNA damage (Monk and Kinross, 1975), but temperature (Matos et al., 2013) and oxidative stress (Figueroa-Bossi and Bossi, 1999; Selva et al., 2009; Los et al., 2010) can also lead to the destruction of the main repressor protein in charge of lysogeny maintenance, triggering excision of the prophage, and its entry into the lytic cycle. In consequence, some antibiotics such as quinolones, because they provoke DNA injury in bacteria, induce prophages (Cowlishaw and Ginoza, 1970). Typical of the intricacy of mobile genetic element regulations, prophages are also induced upon bacterial conjugation, when their DNA enters into a bacterial cytoplasm devoid of phage repressor protein (Jacob and Wollman, 1954). It was also suggested recently that the general quorum sensing molecule N-acyl homoserine lactone (AHL) may induce prophages (Ghosh et al., 2009), but subsequent analysis revealed that the AHL effect was rather to repress phage receptor synthesis, thereby artificially increasing the free phage fraction (Hoyland-Kroghsbo et al., 2013). Even in the absence of apparent signal, prophages are induced at low levels so that laboratory saturated pure cultures (10^9^ bacteria per ml) typically contain between 10^4^ and 10^7^ phage articles per ml (Figure 1B).

Some phage-encoded toxins can be produced by bacteria during lysogeny, but in at least two situations, the Shiga-toxin of enterobacteria and the platelet binding protein (Pbl) of *Streptococcus mitis*, the phage-encoded virulence factors are produced only during the lytic cycle (Mitchell et al., 2007; Tyler et al., 2013). Once released, the Pbl toxin, which is a tail fiber protein, associates with the bacterial surface and permits its adhesion to platelet cells (Mitchell et al., 2007). Some prophages disrupt bacterial genes, which are restored upon phage excision leading to another type of phenotypic change (Rabinovich et al., 2012). Poly-lysogenic strains can therefore exhibit a whole range of phenotypes depending on which prophage is excised, and/or induced up to complete lysis at any time (Asadulghani et al., 2009; Matos et al., 2013). This is illustrated in Figure 1B, showing a population of poly-lysogens under its various potential states: (1) production of background levels of virions, (2) excision of a prophage and appearance of a new phenotype by restoration of the gene that was interrupted by the prophage, (3) adsorbtion of virions at the bacterial surface and use as virulence factors (Mitchell et al., 2007). These general notions are important when considering the impact of phages on ecosystems: the effect of temperate phages on a given microbiota will be profoundly different from that of virulent phages.

#### Phage categories in viral fractions

The viral fraction in the feces of healthy humans appears to be dominated by temperate phages, in sharp contrast with aquatic environments where virulent phages dominate (Reyes et al., 2010). These conclusions are drawn from the abundance, in feces viromes, of integrase gene sequences, the enzyme being responsible for phage integration into the bacterial genome, and therefore constituting a signature of temperate phages: about 1% of reads from feces viromes map to integrases (Reyes et al., 2010), which suggests that at least 50% of phages in the sample are temperate (assuming that a 1 kb-long integrase gene constitutes 2% of a 50 kb temperate phage). In another study of feces viromes, a slightly different analysis also reported that 2.4% of contigs of at least 1 kb possess an integrase gene (Minot et al., 2011). Whether this proportion is altered in the microbiota of patients suffering from diarrhea or other bowel diseases is still unknown. An indication however comes from two reports on coliphages (phages infecting *Escherichia coli*), where feces samples from traveler's diarrhea patients were found to contain higher titers of coliphages compared to healthy individuals stools, and the phages were mostly virulent in the patients, whereas they were mostly temperate in samples from healthy controls (Furuse et al., 1983; Chibani-Chennoufi et al., 2004).

#### Phage diversity

**Figure 2:**
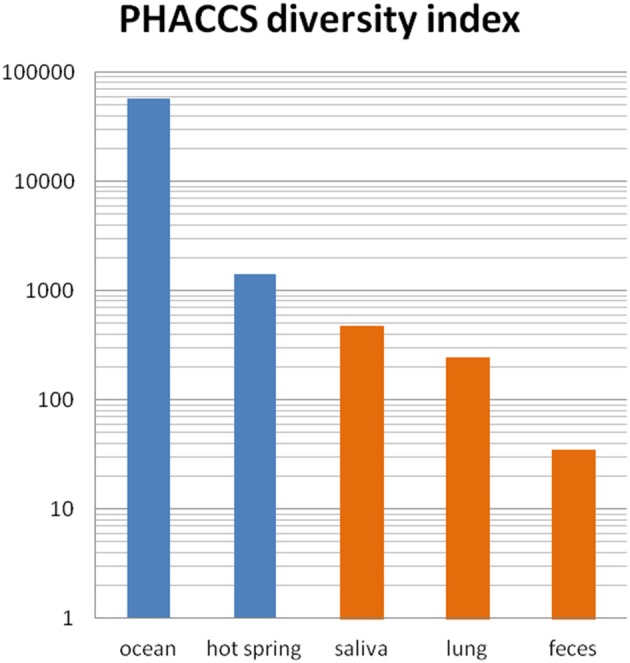
**Phage richness in various viromes**. Scores estimated with PHACCS in five environments are shown (blue, open environments, orange, human samples). PHACCS is a program estimating species richness from contig spectra (Angly et al., 2005). Ocean: mix of four oceans samples (Angly et al., 2006). Hot springs: Yellowstone (Schoenfeld et al., 2008), saliva: average of 5 subjects (Pride et al., 2012), lung: non-cystic fibrosis samples (Willner et al., 2009), feces: median value of 12 samples (Reyes et al., 2010).

Virome studies allow for the first time to apprehend the overall phage genomic diversity, which is even greater than bacterial genomic diversity (Kristensen et al., 2013). It should be noted however, that phage genomic diversity is lower in fecal samples compared to that observed in open environments, as has already been observed for bacterial diversity (Ley et al., 2006). Diversity estimations computed with the same index in five environments are shown in Figure 2. The ratio of virotypes to bacterial phylotypes in the fecal microbiota was estimated to be close to 1 (Reyes et al., 2010). Still, phage genomic diversity is huge in the gut microbiota, and since most metagenomic phage sequences do not map to known phage genes, the functional analysis of viromes remains particularly difficult at present. Further advance in this new field of science will certainly allow refining the picture in the coming years. The analysis of the virome of twins and their mothers has shown that each individual has his own complement of phages, regardless of genetic proximity (Reyes et al., 2010). However, the viral fraction of a given individual remains stable over time in a 1 year study (Reyes et al., 2010). Interestingly, each individual virome is often dominated by one or a few phages (Reyes et al., 2010; Minot et al., 2011).

#### Phage particle quantities

As important as phage diversity, but much less reported, is the number of phage particles encountered in a given sample. Estimates of phage and bacteria concentrations are nevertheless key to the understanding of the phage roles in an ecosystem. “Virus like particles” (VLP) counts are based on fluorescent dye labeling of the samples, followed by microscopic analysis. In aquatic environments where numbers with this technique were first reported (Bergh et al., 1989), VLP counts vary with season and location, but often outnumber bacteria by 3- to 50-fold (Wommack and Colwell, 2000). To simplify a complex reality, bacterial titers in aquatic environments are said to be in the 10^6^/ml range, and VLP around 10-fold more numerous. In the gut of mammals, bacterial density increases along the digestive tract, from an almost sterile situation in the stomach, up to 10^10^–10^11^ per gram of material in the colon or in feces (Berg, 1996). Compared with this dense bacterial population in the terminal part of the gut, VLP concentrations in feces appear to be much lower: estimates of 10^8^ per gram of feces in infant is reported (Breitbart et al., 2008), and another report in adults estimates values between 10^8^ and 10^9^ per gram of feces (Kim et al., 2011). In the bovine ruminal fluid, counts between 10^7^ and 10^8^ VLP/ml have been reported (Letarov and Kulikov, 2009) and references therein. These numbers might be underestimated, as VLP may bind to food particles, bacteria or other surfaces, and therefore may not be efficiently detected. If such low titers of phages in human feces are confirmed in the future, with potentially better techniques, this might indicate that in the lower part of the intestine, the phages encountered are mainly those sporadically produced by bacteria hosting prophages, as is observed upon culturing such bacteria *in vitro* (see above). The situation may be different in the upper parts of the intestine: a study in the mouse intestine reports ranges from 2 to 8 × 10^9^ phages per gram of sample, and finds that phages exceed bacteria by 4-fold (Barr et al., 2013). Moreover, in an experiment designed to test the feasibility of eliminating an *Escherichia coli* population with virulent phages in the mouse digestive tract, it was observed that phages proliferated more in the upper portion of the gut (duodenum) than in the lower part (Maura et al., 2012). This tendency to proliferate in the upper tract may be related to the physiological state of *E. coli*, which multiplies more in this gut section in the animal model used.

#### Importance of the gut environment structure for phage development

Theoretical studies have highlighted that phage dynamics is expected to be radically different if the environment is structured rather than homogeneous, such as in mixed aqueous solutions (Heilmann et al., 2012). This has been validated by experiments comparing phage behavior in liquid medium and semi-solid agar: the phage multiplies more readily on agar (Gama et al., 2013), which may be due to the facilitated bacterium-phage contacts within an agar matrix. Despite the mixing action of intestinal peristalsis, the gut is a structured environment, in which different compartments can be distinguished lengthwise and crosswise. Within each longitudinal segment, at least three compartments can be distinguished: the mucus layer, the lumen, and the food particles, on which bacteria probably grow as biofilms (Brussow, 2013). It was recently shown in mice that some phage particles are 4-fold more concentrated in mucus layer compared to the lumen content (Barr et al., 2013). The authors propose that this situation may favor the host protection against the bacterial species these phages prey on. As for the estimation of VLP concentrations, the technical difficulties linked with the recovery of the distinct tractus compartments should be underlined, and may explain inconsistencies between studies. The mucus forms two layers, the outer one being less structured and more accessible to bacteria and phages, but also more difficult to keep intact. Before the use of the Carnoy protocol (Swidsinski et al., 2005), mucus fixation did not permit recovery of this loose layer of the mucus. Clearly, much remains to be investigated along these lines to better apprehend the distribution of phages in the gut.

In addition to these spatial compartments, water content, pH and oxygen concentration vary greatly according to the gut segment. Most of the water absorption takes place in the large bowel, so that in the distal colon, the lumen content is much less liquid than higher in the GI tract. This parameter may influence the replication of phages, and explain why they tend to replicate better in the upper tract.

### Phages and dysbiosis

Phage infection in the gut may be widespread, as suggested by the presence of abundant phage spacers in the CRISPR (clustered regularly interspaced short palindromic repeat) systems of many human gut bacteria (Stern et al., 2012). But very few data exist so far concerning their role in microbiota functioning and in dysbiosis more specifically. Here we review the ways by which phages can impact a bacterial ecosystem with a focus on gut microbiota. Firstly, phages can impact bacterial ecosystems by predation and multiplication on susceptible bacteria (models 1 and 2, below). Temperate phages can also lyse their previously lysogenic host upon induction (model 3). Alternatively, temperate phages could also impact the ecosystem without killing bacteria, by carrying genes modifying bacterial phenotypes (model 4 in the next section: “emergence of new bacterial strains”).

#### Model 1: “kill the winner” (Figure 3A)

Most of the knowledge concerning the impact of phages on bacterial population through predation comes from studies in the oceans and laboratory models. As phages cannot actively move toward their bacterial preys, they rely entirely on chance to encounter their hosts through random collision, and their ability to infect and reproduce is determined by host density. Both experiments and theory have shown that for a population of phages to multiply, the bacterial population must be above a critical density termed the “replication threshold.” Below this density, the probability of infection is lower than the probability of the phage being lost (Wiggins and Alexander, 1985). As a consequence, phage ability to multiply is possible only if the population of bacterial prey is high, a concept known as the “kill the winner” model, meaning that phage killing concerns only the dominant members of an ecosystem. This model is analogous to classical Lotka–Volterra dynamics that describe predator–prey population dynamics in ecology. Despite the popularity of this concept, only rare demonstrations of this phenomenon exist. Observations of phages/hosts ratios in ocean have confirmed for some bacterial species that phage and host populations indeed oscillate coordinately with time (Hennes and Simon, 1995; Parsons et al., 2012). In the gut however, global studies of phage/bacteria covariation with time have failed to detect any oscillation indicative of a “kill the winner” dynamics, either in humans (Reyes et al., 2010), in pigs (Allen et al., 2011), or in horses (Golomidova et al., 2007). Such dynamics may nevertheless occur but escape observation at the global level, if it concerns limited numbers of sub-dominant species of the gut ecosystem. Moreover, this dynamics could not be detected in monoxenic mice, harboring a microbiota composed exclusively of *E. coli* and coliphages (Ando et al., 1979; Weiss et al., 2009). Several factors could explain this marked difference between gut and aquatic ecosystems, notably the structured semi-solid characteristic of the gut environment, enabling a fraction of the bacterial population to be protected from phages in physical or physiological refuges. Indeed, an attempt at killing a simplified 15-member microbiota by phage cocktails purified from human feces proved successful on two species of this ecosystem during a period of 4–8 days. Later on, the two species were found to have again reached their initial equilibrium concentrations. Contrary to expectations, no phage resistant variants were found in the late growing samples of these strains. This led the authors to suspect, among other possibilities, a phenomenon of refuge, whereby a fraction of the bacteria escaped phage infection (Reyes et al., 2013).

#### Model 2: “biological weapon” or “kill the relative” model (Figure 3B)

Even limited killing of a population of bacteria will cause a partial niche emptying and give an advantage to the competitors capable of exploiting the same niche. Thus bacteria can use their prophages as biological weapons against their main competitors, related bacteria that use the same ecological niche (Bossi et al., 2003; Brown et al., 2006). Rare, spontaneous prophage induction in a small fraction of the lysogenic population can trigger an epidemic among susceptible bacterial competitors, which become factories producing more phage. As the prophage provides immunity to its carrier bacteria against further infection by this phage, the lysogenic bacteria are protected. Thus, by killing competitor bacteria, prophages can indirectly benefit their bacterial host. Virion amplification allows lysogenic bacteria to invade well mixed populations of susceptible bacteria. It has been demonstrated that such amplification is even faster when lysogens are rare at the onset of the experiment (Brown et al., 2006). Sometimes, however, temperate viruses spare recipient cells from death by lysogenizing them, and, for some phages such as λ, the probability of lysogenization increases with the multiplicity of infection. Therefore, phage amplification leads to conflicting predictions about the efficacy of temperate phages as biological weapons: amplification can increase the relative advantage of the lysogenic clone but it also increases the likelihood of saving susceptible cells from death by lysogenization (Gama et al., 2013). Massive lysogenization of susceptible cells occurs in structured environments, under the conditions of substantial phage growth, resulting in a very transient advantage for the lysogenic bacteria (Gama et al., 2013). Competition experiments between lysogen and sensitive strains of *Enterococcus faecalis* in monoxenic mice show a transient and limited, 1.5-fold enrichment of the lysogen over the sensitive strain at 24 h, but no further time point was shown (Duerkop et al., 2012). In summary, in complex microbial communities, where susceptible bacteria are scarce, such “biological weapon” phenomenon is thus likely to confer only a limited advantage to lysogenic bacteria during colonization. It might have an important role however, in protecting the microbiota from invasion by a pathogenic strain, which may be killed by such “weapons,” in keeping with the hypothesis of Barr et al. (2013). Further experimental work is needed to address this question.

#### Model 3: community shuffling model (Figure 3C)

Rather than being beneficial to their bacterial host, prophages can also be detrimental as their induction results in host lysis. Even if spontaneous induction is a rare event with negligible adverse consequence on lysogen fitness, a variety of environmental factors can change induction from a rare and stochastic event to a deterministic process. In oceans, prophages have been compared to molecular time bombs that can be triggered by changes in salinity or by various pollutants (Paul, 2008). In the gut, several inducing agents can potentially trigger prophage induction. For example, some antibiotics at sub-inhibitory concentrations, such as quinolones or beta-lactams, have been shown to induce prophages in several bacterial species such as *E. coli* (Zhang et al., 2000), *Clostridium difficile* (Meessen-Pinard et al., 2012), *E. faecalis* (Matos et al., 2013), or *Staphylococcus aureus* (Goerke et al., 2006; Maiques et al., 2006). Other toxic compounds such as those present in cigarette smoke have been shown to induce *Lactobacillus* prophages *in vitro*. This suggests a causal relation between prophage induction by cigarette smoke and the higher risk for smokers to contract bacterial vaginosis, which is characterized by a reduction of vaginal lactobacilli (Pavlova and Tao, 2000). Oxidative stress is another potent inducer of phages in the gut. *Streptococcus pneumoniae* produces naturally high levels of H_2_O_2_ that were shown, in the nasopharynx, to be responsible for prophage induction and killing of *S. aureus* lysogenic strains, with no adverse effect on non-lysogens (Selva et al., 2009). Inflammation, notably by provoking oxidative stress, might also be responsible for prophage induction. Analysis of colonoscopy biopsies have shown that the mucus of Crohn's disease patients was 30-fold enriched in virus-like particles (3 × 10^9^ VLP/biopsy versus 10^8^ for healthy controls (Lepage et al., 2008). Even in the absence of external signal or inflammation, the *stx2* carrying *E. coli* prophage 933 W is more induced in the gut of monoxenic mice than *in vitro* (Tyler et al., 2013). In *E. coli* and *Lactobacillus* genomes, the vast majority of prophages are defective, indicating an active selection against prophages induction (Martin et al., 2009). This suggests that prior to inactivation, these prophages were induced at levels sufficiently high to favor the emergence of defective variants. Consequences of prophage induction in the gut microbiota can be far reaching. Mills and coworkers have proposed the “community shuffling” model which hypothesizes that upon a stress, prophages can be more induced in mutualistic intestinal bacteria than in opportunist pathogen species, contributing to intestinal dysbiosis by diminishing the ratio of symbionts to pathobionts (Mills et al., 2013). The role of bacteriophages in inflammatory bowel diseases has been suspected for years and several mechanisms can be hypothesized (Riley, 2004), notably the modification of the equilibrium between specific bacterial populations.

### Phages and emergence of new bacterial strains in the gut (Model 4)

**Figure 3:**
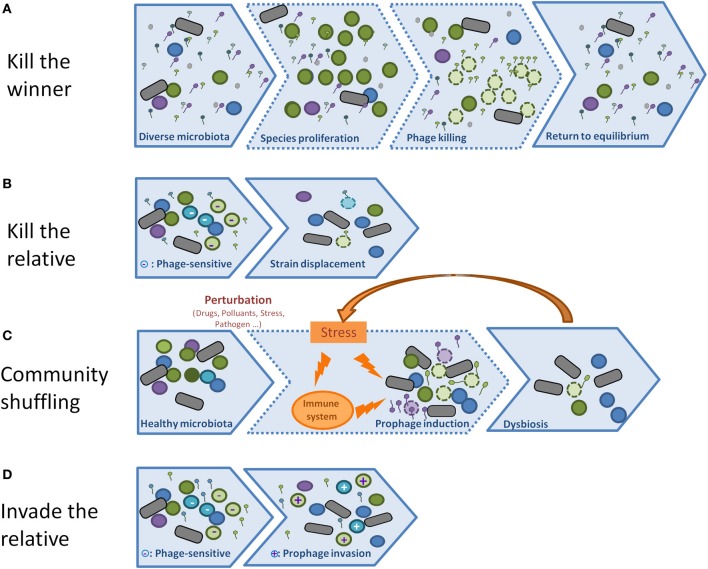
**(A)** In the **Kill the winner** scheme, viruses are more abundant than bacteria, but do not infect them because of their low concentration (step 1). However, if some bacteria overgrow (step 2), and reach the threshold above which phages can absorb and predate them, they will lyse, and the system will shift back to its initial state (3). **(B)** In the **Kill the relative** scheme, phages do not need to be abundant, they are produced by lysogen strains (step 1) and kill their relatives that are not resistant to the phage (bacteria of the same color, but lighter, with a “-”label). The net result is an advantage of lysogenic populations relative to non-lysogens (step 2). **(C)** In the **Community shuffling** scenario, temperate phages act negatively on their host by killing them upon sensing a mild stress that would not have killed a non-lysogen (step 2). A positive feed-back loop may even take place if the massive lysis leads to an host reaction, such as inflammation (orange arrow). The net result is a global displacement of population, and possibly dysbiosis. **(D)** Temperate phages can act profoundly on bacterial population without lysing them: in the **Invade the relative** scheme, the prophage propagates itself by infecting new hosts without lysing them, but by establishing lysogeny (step 2, the + sign indicates new lysogens).

The three above mentioned phage behaviors rely on the capacity of phages to lyse their host. But temperate phages can also propagate by establishing lysogeny into the host they infect, rather than lysing it. Under laboratory conditions, the phage λ tends to enter into the lysis cycle upon infection of an exponentially growing bacterial cell. However, slow growing bacteria, or high multiplicity of infection, are parameters that increase the proportion of lysogens. Whether the lytic or the lysogenic cycle is favored in natural ecosystems is unknown at present, and it will probably depend on the phage, the bacterium, and the ecosystem tested. If lysogeny is favored, the phages should be essentially considered as horizontal transfer vectors that may considerably contribute to the evolution of bacterial strains. An understanding of the relative importance of lysis and lysogeny in various natural environments, in particular that of the human gut, is vital to the understanding of the phage-bacterial ecosystem. For this reason, it is important in the future to design experiments aimed at estimating the lysogenization rate of various phages in natural environments. An important alternative to the above mentioned “kill the relative” (Figure 3B) model is therefore an “invade the relative” situation (Figure 3D), whereby prophages behave as rampant invaders, and thereby contribute to the emergence of new bacterial strains.

Such horizontal transfer might be neutral in terms of selection if the prophage does not change the bacterial phenotype. But data accumulate that suggest on the contrary that prophages, and even defective prophages, do change important bacterial phenotypes, often related to a shift toward pathogenicity, such as increased virulence due to toxin production, or improved host colonization, due to resistance to oxidative stress, bile salts, or biofilm formation (Wang et al., 2010). In many cases, the phage genes responsible for the phenotypes are known. Most of them are “morons” (they bring something “more” to their host), meaning that they have autonomous transcription signals, and are expressed in the lysogenic state (Juhala et al., 2000). Except for a few cases (Shiga-toxin and Pbl), the phage encoded toxins usually belong to this moron category (Brussow et al., 2004; Tinsley et al., 2006). Interestingly, bio-informatics studies suggest that many more morons are present in phage genomes, for which at present no function can be predicted (Tobe et al., 2006).

Recently, several metagenomic studies have underlined the presence of some antibiotic resistance genes in the virome fraction (Allen et al., 2011; Colomer-Lluch et al., 2011; Fancello et al., 2011; Minot et al., 2011; Modi et al., 2013). This contrasts with the knowledge based on complete phage genomes, where antibiotic resistance genes have been scarcely reported. When found, they are associated with transposons (Banks et al., 2003) or insertion sequences (Muniesa et al., 2004) that have invaded the phage genome, a situation often correlated with phage decay (De Paepe et al., 2014). It is also known that some phages encapsidate and transfer large bacterial DNA segments from strain to strain, a process named generalized transduction. A recent survey of various viromes has established that such bacterial sequences were found at low concentrations in most samples extracted from eukaryotic environments (Roux et al., 2013). This process contributes to the spread of antibiotic resistance genes (Muniesa et al., 2004, and references therein). Whether the few antibiotic genes reported in various viromes correspond to these two well known mechanisms, or indicate a new process that has escaped earlier scrutiny remains to be investigated.

A last aspect of the contribution of phages to the emergence of more virulent strains is related to their remarkable capacity of evolution: they mutate and recombine at rates orders of magnitude higher than bacteria (Drake, 1999; Martinsohn et al., 2008; De Paepe et al., 2014). Temperate phages also efficiently exchange sequences with defective phage remnants present in the host in which they multiply (De Paepe et al., 2014). New phages are therefore constantly emerging, with new assortments of gene sequences. Horizontal transfer mediated by phages is therefore more than the simple passage of a given genetic content from one host to the next, but it is likely to contribute actively to the generation of new gene—and genome—combinations.

### Impact of phages on host immunity

Mammals and their intestinal microbes live in a symbiotic relationship. Host tolerance toward its intestinal microbiota is facilitated through physical separation of bacteria and host cells via the mucus layer, by bacterial adaptation to reduce its immunogenicity, and by direct modulation of local host immune responses, through, among other mechanisms, the release of bacterial metabolites that are yet to be fully characterized (Arpaia et al., 2013; An et al., 2014). Our knowledge of how phages contribute to these interactions is still limited.

Among the mechanisms responsible for the recognition of microbial and viral structures are the Toll Like Receptors (for a review see Kawai and Akira, 2011). Ten and twelve TLRs have been described, in mouse and humans respectively, that recognize Pathogen Associated Molecular Patterns (PAMP) including LPS, unmethylated CpG-DNA and flagellin notably.

Viral nucleic acids act as PAMPs and are recognized by multiple TLRs. TLR3, TLR7, TLR8, and TLR9 are involved in the recognition of viral nucleotides such as double-stranded RNA (dsRNA) (TLR3), single-stranded RNA (ssRNA) (TLR7-TLR8), and DNA (TLR9) (Alexopoulos et al., 2010; Blasius and Beutler, 2010; Shi et al., 2011). The hallmark of these nucleic acid-sensing TLRs is that they potently promote the production of type I IFN, in addition to the other inflammatory cytokines that are induced by all TLRs. So far no TLR recognizing specific phage components have been described, but they could have a cell surface or intracellular location. TLR1, TLR2, TLR4, TLR5, and TLR6 are localized on the cell surface and largely recognize microbial membrane components whereas TLR3, TLR7, TLR8, and TLR9 are expressed within intracellular vesicles and recognize nucleic acids (Blasius and Beutler, 2010).

Three main interaction mechanisms between phage and the immune system have been described or suggested:

#### Prophage-mediated changes of recognition patterns of bacteria

Riley hypothesized in 2004 that phages might be involved in inflammatory bowel disease pathogenesis by changing the recognition patterns of bacteria (Riley, 2004). Indeed, it has been demonstrated for *Salmonella, E. coli, Shigella*, and *Vibrio cholerae* that some temperate phages encode enzymes modifying the O-antigen component of the bacterial host outer membrane lipopolysaccharide (LPS), modifying bacterial antigenicity (Verma et al., 1991; Boyd et al., 2012; Brussow, 2013; Davies et al., 2013). Thanks to their prophages, some lysogenic bacteria could then escape the immune system, with radically different outcomes depending on whether the bacteria belong to commensal or pathogenic species.

#### Direct immunity modulation upon phage particles phagocytosis

In addition, phages could be directly recognized by the host and trigger specific modulations. As suggested recently, bacteriophage recognition by antiviral innate immune sensors would happen if cells directly phagocytized phage particles or if phages were delivered to the intracellular environment by phage-producing bacteria (Duerkop and Hooper, 2013). After degradation of the phage particle, phagocytized phage nucleic acids could be sensed by endosomal receptors (TLR7 or TLR9). If intracellular phages are uncoated in the cytoplasm, the released nucleic acids could be sensed by the sensors cGAMP synthase (DNA) or RIG-I (RNA), which signal through stimulator of interferon genes and mitochondrial antiviral-signaling protein, respectively. The cascade leads to NF-κB, IRF3 and IRF7 which promote the transcription of immune effectors such as IFN-β and proinflammatory cytokines.

#### Phages particles interacting with the mucus

Recently, the F. Rohwer team described how the Ig-like domain of the T4 phage Hoc capsid protein could bind mucus (Barr et al., 2013). Because this protein has been detected as abundant in viromes, and highly variable, such interactions are suggested to occur frequently in the human intestine.

A better understanding of the impact of phages on immunity should help for the development of techniques such as fecal transplants, whereby a complete microbiota from an healthy individual is inoculated to a patient with severe colitis (often due to *Clostridium difficile* infection). Fecal transplants have been shown to represent an alternative therapy to restore eubiosis, a balanced microbial ecosystem in the human gut (Allen-Vercoe et al., 2012; Kelly, 2013). However, among the limits that restrain its use, is the fact that phages and viruses are also transferred during the procedure. While inoculum/samples can be typed for the presence of known human and animal viruses, the important biomass, gene pool and potential immuno-modulatory signals represented by phages have yet to be characterized. The impact of phages on the immune system is also actively investigated by teams working on phage therapy. Even though the phages used, and the doses applied, differ significantly from the conditions encountered in natural ecosystems, the effects observed are informative. No major adverse or stimulatory effects have been reported so far, following massive ingestion of phage preparations (Gorski et al., 2012; McCallin et al., 2013; Miernikiewicz et al., 2013).

## Conclusion

Phages play important and diverse roles in all bacterial ecosystems, but their precise impact on the gut microbiota is far from being understood. Present as the major component in most ecosystems, generally outnumbering bacteria by a factor of 10, they appear to be less numerous in the intestine where fecal VLP counts are of the order of 10^8^/g compared to bacterial counts a thousand-fold greater. It is equally interesting that the gut environment differs from most other ecosystems in containing a majority of temperate phages—though virulent phages are found. These temperate bacteriophages presumably result from spontaneous induction, and thus exist mostly as prophages integrated into the bacterial genomes. This finding influences the interpretation of results of experiments to unravel the complexities of bacteriophage-host dynamics in the gut. In the relatively few investigations published to date, little evidence has been found for models invoking a massive killing action of bacteriophages on bacterial populations in the intestinal ecosystem; experiments have shown evidence neither for the cyclical variations of population density typical of a predator-prey relationship, nor for an advantage to lysogenic bacteria in killing non-lysogenic competitors as a result of low-level phage release (“biological weapons”). It might be that the action of bacteriophages on the equilibrium of the gut ecosystem is due to a large extent to their mode of existence as prophages. This conclusion would be in keeping with the importance of prophages in the gut metagenome, where they make up a sizeable proportion (from a few percent to over 10%) of any bacterial genome.

The presence of an active prophage has two major implications for the bacterium. Firstly it may bring a higher sensitivity to antibiotics, some of which are known to induce lytic growth. Other stresses inducing lysogenic bacteriophages may include inflammation caused by travelers' and Crohn's disease, which have been reported to increase counts of VLP in patients' stools. Such induction may result, for example, in “community shuffling” by killing lysogens and thus open niches to colonization by other strains or species. But the most important contribution of bacteriophages to the gut ecosystem is probably their function as vectors of virulence and more generally of adaptation genes, whereby they contribute to a reassortment of virulence factors which results in the creation of new, virulent strains.

Though a number of high-quality investigations have recently been published, interest in the role of bacteriophages in modulating the equilibrium of the gut ecosystem is relatively recent, and the knowledge base correspondingly scant. Indeed, though the existing evidence argues for a more important role of certain of the phage-host interactions exposed in this review, there is little data on which to base a solid hypothesis. In the future, metagenomic approaches, with their potential to determine separately the content in both bacteria and bacteriophages, will certainly provide important information on the role of bacteriophages, and notably on their potential impact on dysbiosis. To elucidate the contribution of the various models presented in this review, a critical point will be to estimate VLP numbers in order to verify whether the global amount of phages increases or not during disease-associated shifts in species composition of the microbiota. If this observation is indeed confirmed, biological experiments will be required in order to determine the direction (or directions) of causality in this association. Studies performed on animal models allow the manipulation of a simplified microbiota, composed of known strains of commensals, pathogens and/or probiotics, containing (or not) known prophages. Such models have already been used to investigate the dynamics of the phage-bacteria ecosystem (killing, lysogenization, passage of the bacteriophage from one strain to another). The development of these models will allow further investigation of the phage bacteria dynamics and, importantly, the monitoring of the effect of various stresses on prophage induction and population composition (possible dysbiosis). Another, relatively simplified gut ecosystem, though not artificial, is the immature microbiota of human newborns. Following the fate of phages and bacteria in this microbiota, less diverse than that of the adult, may lead to a better understanding of the dynamics of phages and bacteria, as well as their potential perturbations during bacterial or viral infections. More generally, virome analyses should be included in the protocols used in longitudinal studies on the microbiota of human cohorts. Apart from DNA-based analyses of the virome, phage proteins or phage-related metabolites may provide important insights. Such components produced by a stressed microbiota may be produced before any shift in the ecosystem composition, and thus constitute markers capable of anticipating dysbiosis.

Though our understanding of the involvement of bacteriophages in the equilibrium of the intestinal microbiota remains partial, the existing data on phages in human or animal-associated microbiota have established their importance, both as vectors of pathogenicity factors in the emergence of virulent strains and as dynamic players in the intestinal ecosystem. Several pioneering investigations have recently begun to decipher the dynamics of phage-bacterium interactions and suggest avenues of research into how they may maintain homoeostasis, favor a shift to dysbiosis, or the overgrowth of opportunist pathogens. Building upon these results, in the next decade the development and optimization of high-throughput ‘omics analyses combined with biological data from constantly refined animal models and comparative volunteer studies will allow important advances in the comprehension of the part played by phages in the gut ecosystem, with the potential for equally great advances in prevention and therapy of intestinal disorders.

### Conflict of interest statement

The authors declare that the research was conducted in the absence of any commercial or financial relationships that could be construed as a potential conflict of interest.
